# Supplementary material for: Patterns of Intron Gain and Loss in Fungi
Source: PLoS Biol. 2004 Nov 30;2(12):e422. doi: 10.1371/journal.pbio.0020422 (PMC532390; doi:10.1371/journal.pbio.0020422)
Supplement: Table S1 — Also available at http://genes.mit.edu/NielsenEtAl/. (4.3 MB ZIP). [file pbio.0020422.st001.zip › NielsenEtAl/html/510.html]

AN7752.1.NCU08291.1.MG01513.1.FG05316.1


```
 CLUSTAL W (1.82) Multiple Sequence Alignments - Introns Inserted


Sequence 1: MG01513.1	438 aa
Sequence 2: FG05316.1	419 aa
Sequence 3: NCU08291.1	421 aa
Sequence 4: AN7752.1	419 aa
Alignment Length: 441 aa
Number Identitical Residues: 268 aa
Alignment Score (without introns) 11865


MG01513.1 	--MVAMRHPARQLCQTFSQTGSSRFAANGLRTLPSSALQTRAFATP-VPPVTQDATGSKG
NCU08291.1	MASMALRLPTAQLCK-AARSSSFRPS------FRCAQQQTRWLATPTTHPVTQKNVG---
FG05316.1 	---MASRISTGQLCRSISQASSHRLA------LPHIAAQRRYLATT-VHPVTQDATGSKG
AN7752.1  	---MALRR-TIALPRMLRSAARIPMG--------ISGEHRRGLATA-VPPVTQDATGSKG
          	   :* *  :  * :    :.    .            : * :**. . ****. .*:..

MG01513.1 	PTAMVFLNMGGPSTTEEVHDFLLRLF0SDADLIPFGPLQKYLAQWISKRRTPKIQGQYAA
NCU08291.1	-TAMVFLNMGGPSTLDEVGDFLSRLF0ADADLIPLGRLQNYLGPLISKRRTPKIQKQYGA
FG05316.1 	PTAMVFLNMGGPSTTDEVGSFLSRLF0ADGDLIPLGPLQNYLGPLISKRRTPKIIKQYSA
AN7752.1  	PTAMVFLNMGGPSTTAEVEDFLSRLF0ADGDLIPLGRLQNYLGPLIAKRRTPKIQRQYAD
          	.*************  ** .** *** :*.****:* **:**.  *:*******  **. 

MG01513.1 	IGGGSPIRKWSEHQNAEMCKLLDKISPETAPHKPYVAFRYANPLTEEMYNRLLADGFGNG
NCU08291.1	IGGGSPIRKWSEYQCEEMCKILDQISPETAPHKPYVAFRYANPLTEEMYKKLLADGFGNG
FG05316.1 	IGGGSPIRKWSEYQNAEMCKILDKISPETAPHKPYVAFRYADPLTEEMYEQLLKDGFGNG
AN7752.1  	IGGGSPIRKWSEYQCAEMCKLLDQLSPESAPHKPYVAFRYAAPLTEEMYAQLLADGFGNG
          	************:*  ****:**::***:************ ******* :** ******

MG01513.1 	KGGRAVAFTQYPQYSCSTTGSSLNELWKWRQRLEGKSAEADGGSSAALSKVNDGTIQWSV
NCU08291.1	RGGRAVAFTQYPQYSCSTTGSSLNELWKWRQRLEGKAGPSEDG--------KDGTIRWSV
FG05316.1 	KGGRAVAFTQYPQYSCSTTGSSLNELWKWRHRLEGKTNKESG----------DGTITWSV
AN7752.1  	KGGRAVAFTQYPQYSCSTTGSSLNELWKWRTRLEGKRANCDVD--------ATGTIQWSV
          	:***************************** *****    . .          *** ***

MG01513.1 	IDRWPVHAGLVEAFAQNIEAKLAEYPEERRNKAVLMFSAHSLPMTVVNRG1DPYPAEVAA
NCU08291.1	IDRWPTHPGLVEAFARNIEEKLAEYPEERRKDVVLLFSAHSLPMSVVNRG1DPYPQEIGA
FG05316.1 	IDRWPNHSGLVEAFAQNIEAKLKEYPEERRKDVVLLFSAHSLPMSVVNRG1DPYPAEVAA
AN7752.1  	IDRWPTHPGLIEAFARNIEDQLKTYPEEKRNSVVLLFSAHSLPMSVVNRG1DPYPAEVAA
          	***** *.**:****:*** :*  ****:*:..**:********:***** **** *:.*

MG01513.1 	TVYGVMQRLGFSNPYRLCWQSQVGPQPWLGPQTSTSVEEYVSKGQKDIVLIPIAFTSDHI
NCU08291.1	TVQAVMQRLNFSNPYRLCYQSQVGPQPWLGPQTQTSVEEYIHKGQKDLVLIPIAFTSDHI
FG05316.1 	TVYAVMQRLGFSNPYRLCWQSQVGPSAWLGPQTSDSVEHYVAKGQKDLVLIPIAFTSDHI
AN7752.1  	TVHAVMQRLNFSNPYRLCWQSQVGPSAWLGAQTSDTVQEYVKRGQTDLVLVPIAFTSDHI
          	** .*****.********:******..***.**. :*:.*: :**.*:**:*********

MG01513.1 	ETLFELDQEVIGDSGHPDTVKRVESLNGSKVFIKALADIAKEHLHSGLACSTQMGLRCPG
NCU08291.1	ETLYELDQEVIGESGHPDTVKRVESLNGNPVFIKALADLAKEHLDSGLACSHQNSLRCPG
FG05316.1 	ETLYELDQEVIADSGSPETVKRVESLNGSPVFIEALADIAKKHLAENKACSKQMGLRCPG
AN7752.1  	ETLYELDLEVMEEANHPG-VKRAESLNGNPIFIQALADLAHDHLRKGELCSLQMTLRCQG
          	***:*** **: ::. *  ***.*****. :**:****:*:.** ..  ** *  *** *

MG01513.1 	CKSERCLESKKFFASQQARFSSA
NCU08291.1	CKSERCAASKKFFAGQEILPGH-
FG05316.1 	CKSERCAESKRFFASQQAGLA--
AN7752.1  	CKSERCLEQKKFFAGQKYASLVQ
          	******  .*:***.*:
```
